# Serum transferrin predicts end-stage Renal Disease in Type 2 Diabetes Mellitus patients

**DOI:** 10.7150/ijms.46259

**Published:** 2020-07-29

**Authors:** Lijun Zhao, Yutong Zou, Junlin Zhang, Rui Zhang, Honghong Ren, Lin Li, Ruikun Guo, Jie Zhang, Fang Liu

**Affiliations:** 1 Division of Nephrology, West China Hospital of Sichuan University, Chengdu, Sichuan, China.; 2 Division of General Practice, West China Hospital of Sichuan University, Chengdu, Sichuan, China.; 3 Division of Pathology, West China Hospital of Sichuan University, Chengdu, Sichuan, China.; 4 Key Laboratory of Transplant Engineering and Immunology, Ministry of Health, Regenerative Medicine Research Center, Chengdu China.

**Keywords:** diabetic nephropathy, end-stage renal disease, transferrin, iron nephrotoxicity

## Abstract

**Background**: To investigate the relationship between serum iron status and renal outcome in patients with type 2 diabetes mellitus (T2DM).

**Methods:** Chinese patients (n=111) with T2DM and biopsy-proven diabetic nephropathy (DN) were surveyed in a longitudinal, retrospective study. Serum iron, total iron-binding capacity, ferritin, and transferrin were measured at the time of renal biopsy. Iron deposition and transferrin staining were performed with renal biopsy specimens of DN patients and potential kidney donors. End-stage renal disease (ESRD) was the end-point. ESRD was defined as an estimated glomerular filtration rate <15 mL/min/1.73 m^2^ or the need for chronic renal replacement therapy. Cox proportional hazard models were used to estimate the hazard ratios (HRs) for the influence of serum iron metabolism on ESRD.

**Results:** During a median follow up of 30.9 months, 66 (59.5%) patients progressed to ESRD. After adjusting for age, sex, baseline systolic blood pressure, renal functions, hemoglobin, HbA1c, and pathological findings, lower serum transferrin concentrations were significantly associated with higher ESRD in multivariate models. Compared with patients in the highest transferrin quartile (≥1.65 g/L), patients in the lowest quartile (≤1.15 g/L) had multivariable-adjusted HR (95% confidence interval) of 7.36 (1.40-38.65) for ESRD. Moreover, tubular epithelial cells in DN exhibited a higher deposition of iron and transferrin expression compared with healthy controls.

**Conclusions:** Low serum transferrin concentration was associated with diabetic ESRD in patients with T2DM. Free iron nephrotoxicity and poor nutritional status with accumulated iron or transferrin deposition might contribute to ESRD.

## Introduction

Diabetic nephropathy (DN) has become the leading cause of end-stage renal disease (ESRD) in Chinese people 1, a phenomenon that parallels the dramatic worldwide rise in prevalence of diabetes mellitus 2. Early detection and better management of DN patients with type 2 diabetes mellitus (T2DM) may delay DN progression to ESRD, lessen its complications, and improve outcome.

Dysregulation of iron homeostasis is a major factor in development of DN. Increased iron stores are associated with diabetes and DN by their effect on inflammation, production of reactive oxygen species, and initiation and propagation of lipid peroxidation 3-5. Conversely, iron deficiency may lead to impaired production of hemoglobin and result in iron-deficient anemia, which is common in patients with DN 6. Disorders of iron homeostasis that occur in chronic kidney disease (CKD) turn anemia management of patients into a complex multifactorial therapeutic task 7. New erythropoiesis-stimulating agents (ESA) such as darbepoetin 8 and others in development such as the continuous erythropoietin receptor activator 9 have longer half-lives than recombinant human erythropoietin, enabling CKD outpatients to require less frequent ESA administration. Despite the foregoing advances in CKD-associated anemia management, the one unresolved issue is the prognostic value of iron status in CKD patients. Locatelli *et al.* proposed serum ferritin and transferrin saturation measurements as diagnostic markers of iron status 10. Choe *et al.* found that abnormal iron status increased the mortality of patients with diabetes 11. High serum ferritin values were associated with mortality in hemodialysis patients 12, 13. Although the effect of iron status in clinical outcome has been examined extensively in the dialysis population 12, 14, there are only limited data that describe the association between iron indices and renal outcomes in the pre-dialysis population with T2DM and associated DN. Iron increases insulin resistance, and iron is related to oxidative stress in T2DM 15. Insulin resistance and oxidative stress are risk factors for kidney progression in T2DM. Thus, it is plausible to postulate that abnormal iron homeostasis was associated with renal outcomes in patients with T2DM and associated DN.

In this retrospective cohort study, we found that reduced serum transferrin was associated with diabetic ESRD. Free iron nephrotoxicity and poor nutritional status with accumulated iron or transferrin deposition might be mechanisms that contribute to DN progression.

## Materials and Methods

### Design, Setting, and Participants

**Figure 1:**
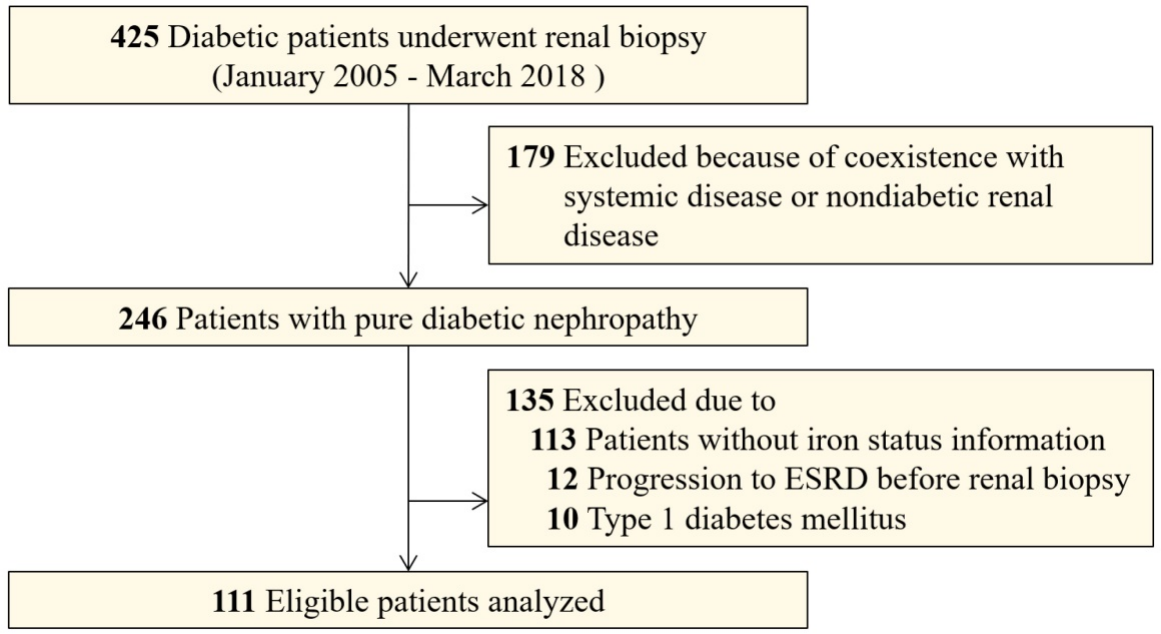
Flowcharts of patients in this study.

To investigate the association between iron status and renal outcome, this longitudinal, retrospective study included patients with T2DM and associated DN who underwent percutaneous renal biopsy from January 2010 to March 2018 at the West China Hospital of Sichuan University. All patients provided informed consent and the Institutional Review Boards of Sichuan University approved this study. The indications for renal biopsy were T2DM patients with renal damage who lacked absolute contraindications, especially T2DM patients without diabetic retinopathy, or with obvious glomerular hematuria and/or short diabetic duration, or with sudden onset overt proteinuria 16. We used the American Diabetes Association criteria for T2DM diagnosis 17. DN was defined based on the 2015 standard of An *et al.* 18 , and DN was diagnosed by at least two renal pathologists and/or nephrologists based on Renal Pathology Society (RPS) classification 19. Adult patients with T2DM and biopsy-proven DN were eligible for this study. Exclusion criteria were coexisting nondiabetic renal diseases (NDRD) such as IgA nephropathy or systemic diseases, especially anti-neutrophil cytoplasmic antibody (ANCA) that are associated vasculitis, anti-glomerular basement membrane (GBM) disease and lupus nephritis, non-T2DM, progression to ESRD before renal biopsy, and patients who had incomplete iron status information (Figure 1). In sum, we enrolled 111 patients who had DN as the only glomerular disease diagnosis and for who completed iron status data were available.

For tissue transferrin and iron staining, renal biopsy specimens were collected from ten potential kidney donors (healthy controls) and age, sex-matched patients with T2DN in our cohort. Table S1 displays their clinical characteristics.

### Clinical and pathologic information

Clinical and pathologic data were abstracted from electronic medical records at the time of renal biopsy. Data included age, sex, body mass index (BMI), smoking status, presence of diabetic retinopathy, and use of renin-angiotensin aldosterone system (RAAS) blockade or ESA. Smoking status was determined at the time of biopsy. Diabetic retinopathy was defined as existence of microaneurysms, retinal dot, blot hemorrhage, or neovascularization in the retina 20. In addition, laboratory data at the time of biopsy were obtained from the medical records. We evaluated the estimated glomerular filtration rate (eGFR) using the creatinine-based Chronic Kidney Disease Epidemiology Collaboration equation 21. Hematuria was defined as more than five erythrocytes per high-power field in at least two of three consecutive urine tests without urinary infection and no urinary tract malignancy or stone 22, 23. Treatment was defined as the use of the RAAS blockade, statins, and ESA for more than half of the follow-up period. Patient follow-up examinations were performed 2-4 times per year based on the patient's individual condition. Renal outcome was defined by the progression to ESRD, which was defined as eGFR <15 mL/min/1.73 m^2^ or the need for chronic renal replacement therapy 24. All patients were followed until March 31, 2019.

### Markers of serum iron status

Baseline iron status at the time of renal biopsy was measured with standard protocols at West China Hospital. Serum iron concentrations and iron saturation ratios were used as indices of iron stores. Total iron-binding capacity (TIBC) was represented serum transferrin level. Transferrin was measured by chemiluminescence immunoassay, and serum ferritin was measured with an immunoradiometric assay. Serum iron and total iron-binding capacity were measured with a modified automated AAII-25 colorimetric method. Transferrin saturation (TSAT) was calculated as (iron/TIBC) *100%.

### Histological analysis

Renal biopsy tissues for light microscopy, immunofluorescence, and electron microscopy were prepared by standard procedures at West China Hospital and examined by expert nephropathologists. Sections for light microscopy were stained with hematoxylin-eosin, periodic acid-Schiff, Masson's trichrome, and periodic acid-Schiff silver methenamine. The original biopsy results from immunofluorescence microscopy and electron microscopy were used to confirm the diagnosis of pure DN. All light microscopy pathological features evaluated in this study were based on the RPS DN classification 19.

### Immunohistochemical staining

Paraffin-embedded kidney tissue samples were sectioned (3 μm) and deparaffinized. Immunohistochemistry was performed as described 25. Briefly, tissue samples were treated with 3% hydrogen peroxide in the dark to block endogenous peroxidase and then subjected to antigen retrieval by heated citric acid. The tissue samples were incubated with 3% normal goat serum (ZLI-9021, Zhongshan, China) at 37°C for 1 hour. Without washing, they were incubated with mouse monoclonal anti-transferrin antibody (sc365871, Santa Cruz, USA) at 4°C overnight and then incubated with an HRP-conjugated goat anti-mouse antibody (8125, CST, USA) at 37°C for 1 hour. Antibody binding was detected by incubating with a fresh mixture of diaminobenzidine (DAB, 8059S, CST, USA) according to the manufacturer's instructions. Slides were counterstained with Mayer's Hematoxylin, dehydrated, and mounted with Entellan (107960, Merck, Germany). Images were captured with a Nikon DXM 1200/NIS-Elements mounted on a light microscope (Nikon Eclipse E600, Shanghai, CHN) and analyzed using Image Pro Plus 6 (IPP software, Houston, TX). Staining intensity was assessed by Image Pro Plus 6 with five randomly selected images per biopsy after correction for background staining.

### Iron detection

For iron detection, paraffin-embedded kidney tissue samples were cut into 4-μm-thick sections and deparaffinized. Then, slides were incubated in the dark with Perl's blue staining reagent according to the kit's instructions (G1422, Solarbio, China). Slides were counterstained with 0.1% nuclear fast red.

### Statistical analysis

Continuous variables were expressed as the mean and standard deviation (SD) with symmetric distribution or as the median and interquartile ranges (IQR) with the asymmetric distribution. Categorical variables were expressed as counts and percent. Differences between continuous variables were analyzed by one-way ANOVA, followed by least significant difference (LSD) tests for multiple comparisons, or the Kruskal-Wallis H test, as appropriate. Categorical variables were analyzed using the Chi-square test or Fisher's exact test. We used Spearman's correlation analysis to assess correlations between serum iron status parameters and clinical findings.

Survival cures of quartiles of serum iron, TIBC, transferrin, ferritin, and TSAT levels were obtained by Kaplan-Meier methods with a log-rank test. Univariate and multivariable Cox proportional hazard models were used to estimate the hazard ratios (HRs) for ESRD 26. One patient was lost to follow-up and only the baseline clinical and pathologic data were analyzed for that patient. Data for 24 h proteinuria were missing for nine individuals (these nine patients were included in multivariable analyses). We first examined the differences in clinical parameters between patients with or without missing values to check whether the values were missed randomly. We then used multiple imputation methods for multivariable models. The proportional hazard assumption in Cox models was tested to check whether the dataset satisfied the basic assumptions of Cox analyses. The Cox proportional hazards model was used to calculate hazard ratios (HRs) and 95% confidence intervals (CIs) for ESRD. In the two Cox proportional hazards models, each HR was adjusted for age, sex, systolic blood pressure, eGFR, proteinuria, hemoglobin and HbA1c at the time of renal biopsy. In “Model 2”, we adjusted each HR for the aforesaid factors plus all pathological parameters as categorical variables. The clinical covariates were selected as potential confounders because of their significance in univariate analysis or on the basis of biological plausibility. Age and sex were chosen on the basis of biological plausibility, and HbA1c was selected to represent previous glycemic control. Proteinuria, eGFR, serum albumin, and hemoglobin were significant in univariate model. Parameters with *P*<0.05 in “Model 2” were considered significant prognosis predictors. Further, to determine the best predictors of ESRD, we used receiver operating characteristic (ROC) curve analysis with clinical/pathological variables, iron status parameters, or their combinations. The area under the curve (AUC) was calculated for each model 27.

All statistical analyses were completed using SPSS software (version 20.0, Chicago, IL, USA) and Stata SE (version 14.0, StataCorp LLC, College Station, TX, USA). Statistical tests were considered significant at *P*<0.05. Figures were constructed using Graph-Pad Prism 8.0 Software (Graph Pad Software, USA).

## Results

### Baseline characteristics

A total of 111 patients with patients with T2DM and biopsy-proven DN from West China Hospital were retrospectively included (Figure 1). The clinical characteristics of the patients with and without iron status data are displayed in Table S2. The patients enrolled in this study had a higher prevalence of hypertension than those for whom iron status data was not available. The patients with iron status had no significant differences in eGFR, proteinuria, and hemoglobin concentration than those without iron status data. A summary of the demographics and baseline characteristics of the 111 patients enrolled in the study is shown in Table 1. The proportion of male patients was 64.9% (n=72). The median baseline eGFR was 51.5 mL/min/1.73 m^2^ (interquartile range [IQR], 34.6-66.8) and the median baseline 24 h proteinuria was 4.64 g/day (IQR, 2.51-8.37). Sixty-two (55.9%) patients had diabetic retinopathy and 66 (59.4%) patients had diabetic neuropathy. The baseline pathological characteristics of patients enrolled in this study were displayed in Table 2. According to RPS classification, there were four (3.6%) patients in class I, 18 (16.2%) patients in classes IIa and IIb, 65 (58.6%) patients in class III, and 24 (21.6%) patients in class IV.

During a mean follow-up of 30.9 months, 66 (59.5%) patients progressed to ESRD. Compared with patients without ESRD, ESRD patients had higher levels of baseline creatinine and proteinuria, lower baseline eGFR, and lower concentrations of serum albumin, hemoglobin, serum iron, TIBC, ferritin, and transferrin (Table 1). There was no significant difference between the prevalence of diabetic retinopathy or diabetic neuropathy in patients with or without ESRD.

### Associations between iron status and ESRD

Of the 111 patients, the median baseline serum iron, ferritin, TIBC, and transferrin concentrations were 10.17 µmol/L (IQR, 8.00-15.70), 311.0 ng/mL (IQR, 168.9-555.1), 35.4 µmol/L (IQR, 29.32-42.2), 1.38 g/L (IQR, 1.15-1.65), respectively. The median TSAT was 30.9 % (IQR, 21.5-39.7). Kaplan-Meier curves showed that serum transferrin and TIBC, instead of serum iron, ferritin, and TSAT, were significant for ESRD (Figure S1). When stratified by quartiles of serum transferrin, the 5-year renal survival rates were 0% for Q1 (≤1.15 g/L), 13.5% for Q2 (1.15-1.38 g/L), 7.3% for Q3 (1.38-1.65 g/L), and 50.9% for Q4 (≥1.65 g/L), respectively (Table S3).

**Figure 2:**
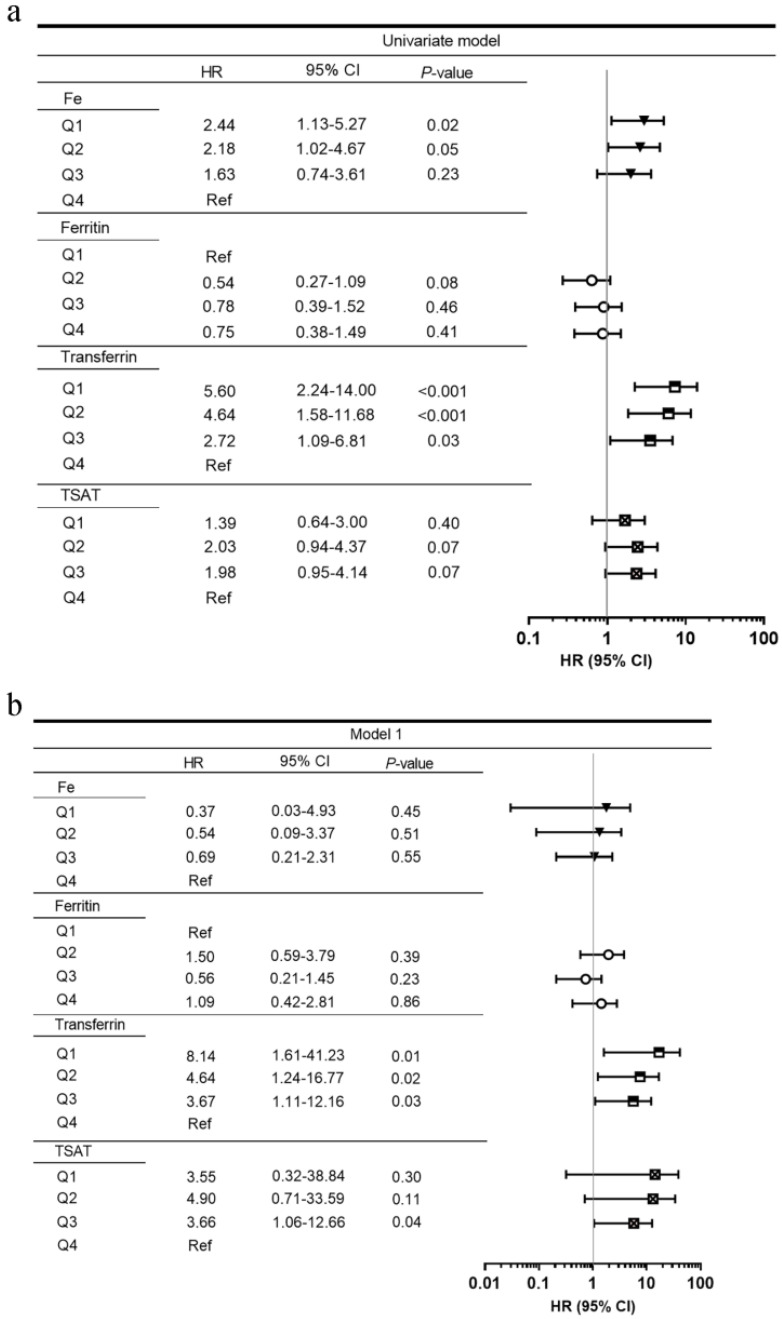

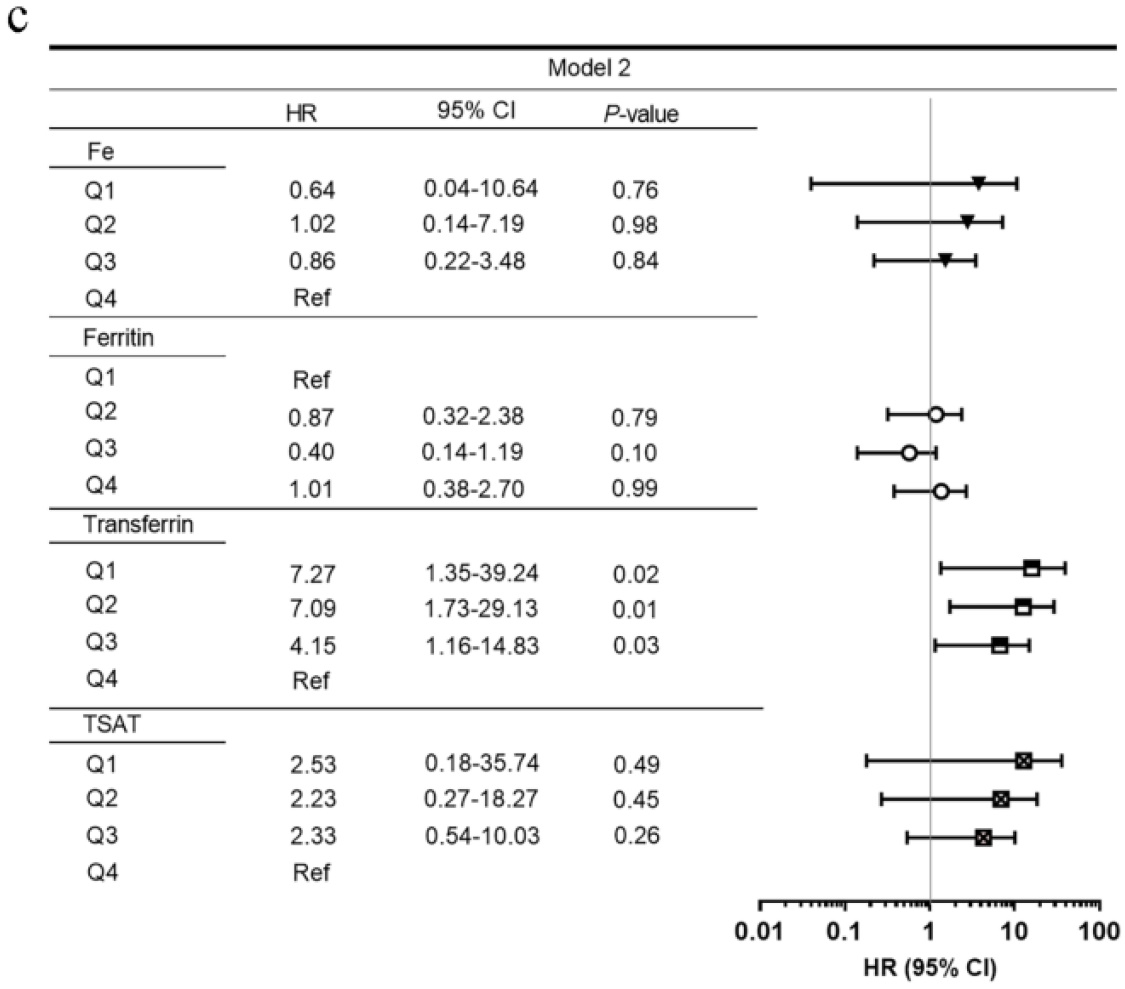
Univariate (**a**) and multivariate (**b, c**) Cox proportional hazard models by serum iron status at the renal endpoint. Model 1: adjusted for age, sex, estimated glomerular filtration rate, proteinuria, hemoglobin, and HbA1c. Model 2: adjusted for the above plus pathological parameters including Renal Pathology Society diabetic nephropathy class, tubular atrophy and interstitial fibrosis, interstitial inflammation, arteriosclerosis, and arteriolar hyalinosis. **Abbreviations:** HR, hazard ratio; CI, confidence interval; TIBC, total iron-binding capacity; TSAT, transferrin saturation; Ref, reference.

Figure 2 shows the adjusted HRs of serum iron status markers for renal survival. After adjusting for clinical parameters (age, sex, SBP, baseline eGFR, proteinuria, hemoglobin, and HbA1c) and pathological findings, lower serum transferrin levels were incrementally associated with higher ESRD in multivariate models. Compared with transferrin Q4, the HRs for transferrin Q1, Q2, and Q3 were 7.36 (95% CI, 1.40-38.65), 7.54 (95% CI, 1.85-30.77), and 3.69 (95% CI, 1.10-12.32). The lowest TIBC quartile (Q1, ≤29.32 µmol/L) was also associated with ESRD (HR 3.88, 95% CI 1.14-13.25) when compared with the highest quartile (Q4, ≥42.2 µmol/L). Moreover, eGFR was an independent risk factor for predicting ESRD (HR 0.84, 95% CI 0.72-0.99).

Figure S2 displays the ROC AUC for the prediction of ESRD by clinical parameters. Among the serum iron status parameter models, serum transferrin had the largest AUC (Figure S2a). Compared with the traditional kidney functional biomarkers, serum transferrin had higher AUC (Figure S2b). Moreover, serum transferrin had higher AUC than any RPS pathological parameter (Figure S2c). Because eGFR, proteinuria, and hemoglobin were associated with ESRD in univariate Cox proportional hazard models, we created a clinical model with those parameters. The model that included transferrin had a larger AUC than the model with only clinical covariates (Figure S2d).

### Clinical characteristics stratified by transferrin

Table 3 lists baseline clinical characteristics of patients divided by quartiles of transferrin. Apparently, patients with lower serum transferrin had lower concentrations of hemoglobin, albumin, uric acid, serum iron and TIBC, and higher levels of proteinuria, TSAT, and ferritin. There seemed to be no difference in age, baseline eGFR, and HbA1c among the four groups. In the Q4 group, 29.6% of patients entered ESRD, which was a significantly lower percent than those in Q1, Q2, and Q3 groups (76.9% in Q1, 73.1% in Q2, 59.4% in Q3, respectively). As for pathological findings, only RPS classification differed significantly among the four groups. Compared with Q4, patients in Q1, Q2, and Q3 group had higher percent of class III (76.9% in Q1, 69.2% in Q2, 50.0% in Q3, and 40.7% in Q4, respectively, Table 4).

Spearman's correlation analysis showed that baseline serum transferrin positively correlated with baseline eGFR (*r*=0.21, *P*=0.03), serum hemoglobin (*r*=0.39, *P*<0.001) and albumin (*r*=0.69, *P*<0.001) but negatively correlated with proteinuria (*r*=-0.42, *P*<0.001) (Figure S3).

### Tissue iron and transferrin staining

**Figure 3:**
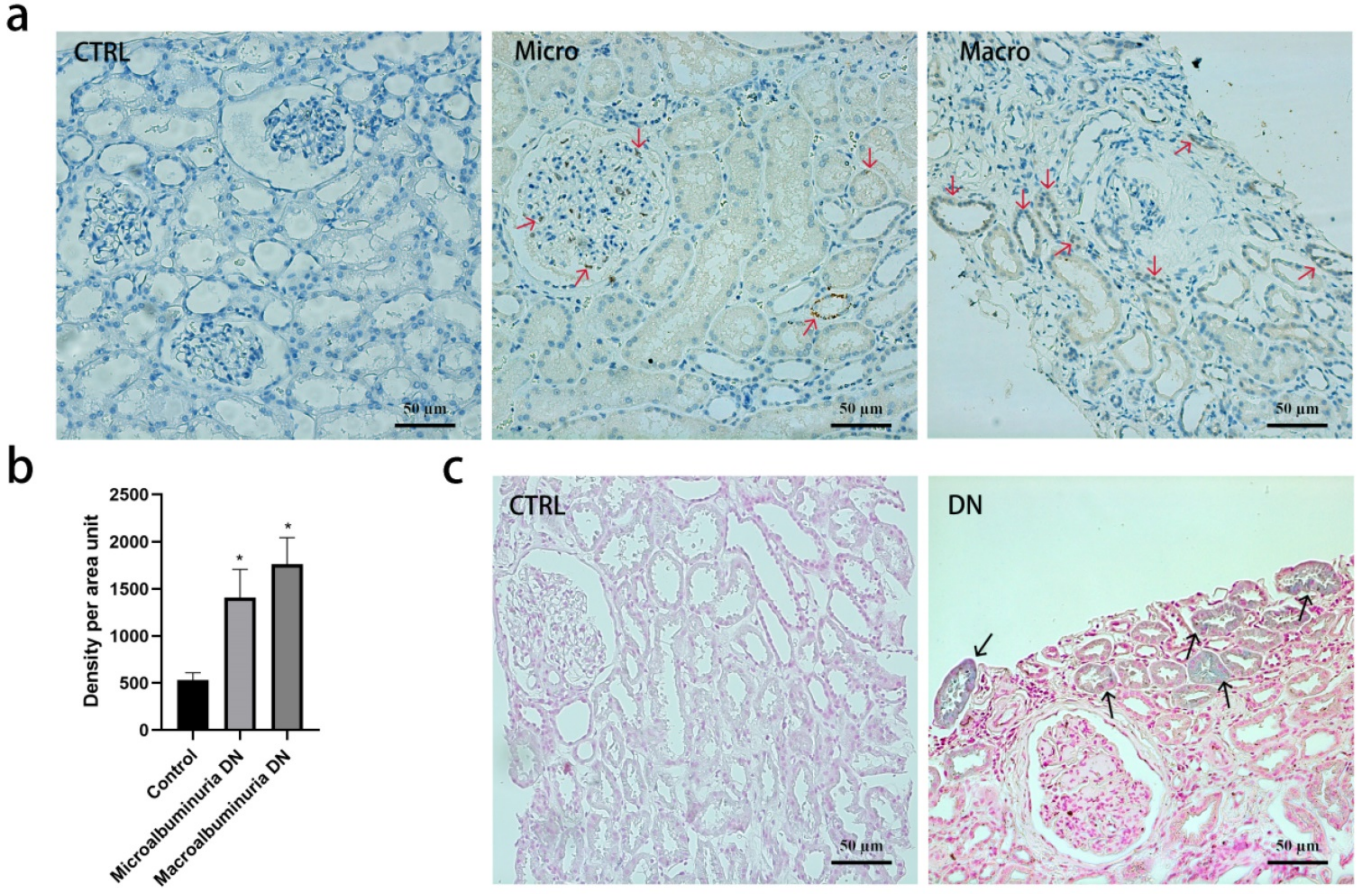
Transferrin and iron staining in kidney biopsy specimens. (**a**) Tissue transferrin (red arrows) by immunohistochemistry in normal kidney tissue, diabetic kidney tissue with microalbuminuria or macroalbuminuria. 200×, Bar=50 μm. (**b**) Quantification of the transferrin staining shows a significant increase in diabetic kidney tissue compared with normal kidney tissue (*p <0 001, compared with control group). Experiments were performed with ten participants for each group, and the results are expressed as mean ± SD. (**c**) Tissue iron deposition (black arrows) by Perl's staining in normal kidney tissue and diabetic kidney tissue. 200×, Bar=50 μm.

Immunohistochemistry revealed that transferrin was absent in normal kidney tissue but positive in DN kidney biopsies (Figure 3a and 3b). Staining of transferrin was strongly positive in the tubular epithelial cells but glomerular staining was less intense in DN. Perl's staining demonstrated that iron was deposited in a granular pattern in the tubular epithelial cells in DN, but not in controls (Figure 3c).

## Discussion

Our results demonstrated that serum transferrin was an indicator for predicting ESRD in patients with T2DM and biopsy-proven DN, independent of other clinical features and pathological findings. Moreover, in DN, we detected iron deposition and transferrin staining more intensely in the tubular epithelial cells compared with normal controls. To our knowledge, this study is the first to reveal the associations between iron status and renal outcomes in patients with T2DM.

Serum transferrin, a glycoprotein with two-iron binding domains, is the most important molecule for transporting iron into cells. Produced mainly in the liver, transferrin has diverse functions such as iron transport across intestinal mucosa, intracellular iron transport, and, by chelating free iron, providing non-specific immunity against microorganisms 28, 29. Previous study showed that low serum transferrin concentration was associated with liver transplantation and/or death 30. But the relationship between transferrin and ESRD was unclear. The low concentrations of serum transferrin in patients with DN might be related to increased urinary transferrin excretion and iron deposition in the kidney. One of the proposed mechanisms of renal damage by transferrin is related to free iron toxicology. Zhang *et al.* suggested that transferrin-bound iron in the circulation is filtered by the pathological glomerulus into the tubular lumen 31, where it binds to transferrin receptors on the surface of tubular cells and is internalized by endosomes, ultimately leading to release of iron 7. Our assays revealed that iron was deposited heavily in tubular cells, thus, providing a source of iron that could act on renal tubular cells. In a diabetic circumstance, cytokines cause an increase in transferrin receptors on the cell surface, favoring tissue accumulation of transferrin and deposition of iron 32. Iron nephrotoxicity is due to 1) the production of cell-damaging reactive radicals by Fenton reactions, and 2) ferroptosis, programmed cell death triggered by iron 33, and 3) iron-induced RAS activation by upregulation of intra renal renin expression 34.

Another potential mechanism of renal injury by transferrin was the direct effect on proximal tubular cells or podocytes. In the early stages of DN, Gonzalez *et al.* found transferrin preferentially deposited in the cytoplasm of glomerular podocytes from diabetic patients 35. The iron liberated from transferrin may contribute to the diabetogenic effect on the kidney. The increased transferrin upregulated complement C3 synthesis, and endothelin-1 and monocyte chemoattractant peptide-1 expression in cultured proximal tubular cells 36, which led to cell death.

In addition to its iron handling function, Reeds *et al.* proposed that transferrin concentration could be used to assess nutritional status 37. Neyra *et al.* reported that serum transferrin had a shorter half-life than albumin, thus, serum transferrin concentration is theoretically a more sensitive marker of early protein depletion 38. In our study, we observed that serum transferrin level was positively correlated with serum albumin and hemoglobin, which suggested that patients with lower transferrin had poor nutritional status and, in turn, they were more susceptible to infection.

TIBC was considered to represent serum transferrin levels 39, 40. Although correlation between TIBC and transferrin is generally good, the reported conversion factors between the two analytes show large differences. Because iron binds to other plasma proteins (mainly albumin), TIBC methods generally overestimate transferrin iron-binding capacity 41. We found that only the lowest TIBC quartile was associated with ESRD.

Previous epidemiology studies showed that low ferritin and high transferrin saturation were associated with better renal outcomes in patients with CKD or hemodialysis 42-44. However, we did not find these relationships in our cohort. Ferritin is the main iron storage protein, and ferritin concentration is a common primary parameter for diagnosing absolute iron deficiency. Ferritin is also regulated by acute-phase proteins. The chronic inflammation in DN could contribute, to some extent, to increased ferritin concentration 45. In addition, the increase in serum ferritin that occurs during infection, in liver disease, and which is associated with malignancies, may hinder assessment of iron in DN with the concurrent presence of the foregoing conditions 46. Thus, the relationship between ferritin and renal outcomes in patients with CKD should be viewed with caution for patients with DN. TSAT reflects iron transport and sequestration, but it is also associated with inflammatory markers in DM. Although serum ferritin and transferrin saturation are easily attainable, they exhibit large biological variability that, contributes to inaccurate prediction of ESRD in DN.

There are several limitations to this study. First, due to the retrospective observational design, selection bias was inevitable and the sample size was limited. Nevertheless, we included in our analyses essentially all patients with biopsy-proven DN and complete iron status data who met the inclusion criteria (Figure 1). The clinical characteristics distributed similarly between patients with and without complete iron status data. The association of the serum transferrin with ESRD remained robust even after adjustment of multiple confounding factors. Second, in this association study, we could not discern whether altered iron homeostasis was a consequence or a driver of disease progression in patients with DN. Previous studies supported the conclusion that iron and iron-containing molecules overload the kidney and cause direct injury to renal tubular cells in *vitro* 47, 48 and in* vivo* 49. In addition, in animal models of DN, iron chelators reduced the severity of kidney injury 50-52. On the basis of current evidence, we propose that the excess tissue iron may cause development and progression of kidney disease 53. Third, for this single-center study, we recruited only biopsy-proven DN patients, a condition that limited generalization of the prognostic value of low serum transferrin concentration for progression to ESRD. Still, the patients in our study included each stage of CKD with a wide range of diabetic duration. Moreover, all patients had undergone renal biopsy and had accurate pathology data. The comparisons of pathological parameters and iron status data for renal prognosis, which could not be obtained in the generalized diabetic population, provided stronger evidence for the conclusion of this study. The results in this study suggested that iron status should be monitored during all stages of chronic kidney disease.

In summary, we showed that serum transferrin predicted kidney-free survival independent of other iron status markers in biopsy-proven DN patients. Simultaneous changes in the same direction for serum transferrin and eGFR predicted a worse renal outcome. Therefore, in clinical settings, both low serum transferrin concentration and low eGFR can be of high diagnostic value in concomitantly predicting ESRD. Our findings emphasized that the availability of catalytic iron or iron that was available to participate in free radical reactions was a prerequisite for iron toxicity. Interventional trials are necessary to examine strategies to modulate the effect of iron metabolism on ESRD.

## Supplementary Material

Supplementary figures and tables.

## Figures and Tables

**Table 1:** Baseline clinical characteristics of individuals with and without ESRD

Characteristics	Total (n = 111)	ESRD + (n = 66)	ESRD - (n = 45)	*P* value
Age, mean (SD), y	51 (9)	51 (9)	52 (9)	0.42
Sex, Male, n (%)	72 (64.9)	42 (63.6)	30 (66.7)	0.84
Smoking, Never/Ex/Current, (n)	64/20/27	38/13/15	2026/7/12	0.80
History of Hypertension, n (%)	105 (94.6)	62 (93.9)	43 (95.6)	0.71
BMI, mean (SD), kg/m2	25.1 (3.38)	24.25 (2.99)	26.12 (3.59)	0.02
SBP, mean (SD), mmHg	149 (23)	150 (23)	147 (24)	0.44
DBP, mean (SD), mmHg	87 (12)	88 (11)	86 (13)	0.56
MAP, mean (SD), mmHg	108 (14)	109 (13)	106 (15)	0.44
Duration of diabetes, median (IQR), months	96 (36-132)	90 (36-132)	96 (36-132)	0.79
History of DR, n (%)	62 (55.9)	39 (59.1)	23 (51.1)	0.44
History of Diabetic neuropathy, n (%)	66 (59.4)	40 (60.6)	28 (57.8)	0.37
HbA1c, median (IQR), %	6.9 (6.2-8.1)	6.9 (5.9-8.1)	7.2 (6.3-7.9)	0.91
FPG, median (IQR), mg/dL	126.64 (91.33-169.88)	118.63 (89.17-174.56)	130.43 (99.08-168.26)	0.67
Hemoglobin, mean (SD), g/L	104 (24)	96 (16)	115 (30)	<0.001
Serum albumin, mean (SD), g/L	31 (7.9)	27.8 (6.5)	35.6 (7.6)	<0.001
CKD stage, 1/2/3/4, (n)^†^	16/23/48/24	6/14/26/20	10/9/22/4	0.02
BUN, median (IQR), mg/dL	25.99 (18.74-37.82)	30.94 (19.36-40.2)	24.45 (17.23-32.49)	0.10
Serum creatinine, median (IQR), mg/dL	136.6 (107-197.6)	145.5 (115-241)	124 (88-149)	<0.001
eGFR, median (IQR), mL/min/1.73 m^2^	51.5 (34.6-66.8)	45.7 (27.6-62)	54.2 (44-80.5)	0.01
24 h proteinuria, median (IQR), g/d	4.64 (2.51-8.37)	5.97 (3.78-8.93)	2.81 (1.43-5.95)	0.01
Hematuria, n (%)	60 (54.1)	39 (59.1)	21 (46.7)	0.20
UA, mean (SD), mg/dL	6.3 (1.4)	6.14 (1.17)	6.53 (1.68)	0.19
Triglyceride, mean (SD), mg/dL	170.67 (115.75)	157.57 (76.26)	189.88 (155.79)	0.20
Cholesterol, mean (SD), mg/dL	204.21 (69.66)	216.2 (74.86)	186.61 (57.61)	0.03
HDL, mean (SD), mg/dL	54.9 (21.39)	58.03 (24.33)	50.3 (15.28)	0.04
LDL, mean (SD), mg/dL	121.82 (55.65)	130.88 (61.12)	108.52 (43.82)	0.04
No. of hypertensive drugs, median (IQR)	2 (1-2)	2 (1-2)	2 (1-2)	0.12
RAAS inhibitor, n (%)	85 (76.6)	47 (71.2)	38 (84.4)	0.11
Statins, n (%)	63 (56.8)	37 (56.1)	26 (57.8)	0.86
ESA, n (%)	9 (8.1)	7 (10.6)	2 (4.4)	0.24
Fe, median (IQR), mmol/L	10.17 (8.00-15.70)	9.45 (6.64-12.59)	12.8 (8.9-18.06)	0.01
TIBC, median (IQR), mmol/L	35.4 (29.32-42.2)	32.5 (27.4-36.83)	40.76 (31.14-45.77)	<0.001
TSAT, median (IQR), %	30.9 (21.5-39.8)	30 (21.9-37.8)	31.7 (20.8-44.3)	0.31
Ferritin, median (IQR), ng/mL	311 (168.9-555.1)	288.3 (149.7-535.1)	314.6 (176.8-547.5)	0.04
Transferrin, median (IQR), g/L	1.38 (1.15-1.65)	1.27 (1.07-1.44)	1.59 (1.22-1.79)	<0.001

Data are presented as the mean (standard) for continuous variables with symmetric distribution, median (25^th^-75^th^ percentiles) for continuous variables with asymmetric distribution, or percent for categorical variables. ^†^ CKD stage1: eGFR≥90 mL/min/1.73 m^2^; stage 2: eGFR 60-89 mL/min/1.73 m^2^; stage 3: eGFR 30-59 mL/min/1.73 m^2^; stage 4: eGFR 15-29 mL/min/1.73 m^2^. **Abbreviations:** SD, standard deviation; IQR, interquartile range; BMI, body mass index; SBP, systolic blood pressure; DBP, diastolic blood pressure; MAP, mean blood pressure; DR, diabetic retinopathy; CKD, chronic kidney disease; HbA1c, hemoglobin A1c; FPG, fasting plasma glucose; BUN, blood urea nitrogen; eGFR, estimated glomerular filtration rate; UA, uric acid; HDL, high-density lipoprotein cholesterol; LDL, low-density lipoprotein cholesterol; RAAS, renin-angiotensin-aldosterone system; ESA, erythropoiesis-stimulating agent; TIBC, total iron-binding capacity; TSAT, transferrin saturation; ESRD, end-stage renal disease.

**Table 2:** Pathological characteristics of individuals with and without ESRD

Characteristics	Total (n = 111)	ESRD + (n = 66)	ESRD (n = 45)	*P* value
**RPS classification^†^, n (%)**				0.01
I	4 (3.6)	0 (0)	4 (8.9)	
IIa	9 (8.1)	2 (3.0)	7 (15.6)	
IIb	9 (8.1)	5 (7.6)	4 (8.9)	
III	65 (58.6)	45 (68.2)	20 (44.4)	
IV	24 (21.6)	14 (21.2)	10 (22.2)	
**IFTA^†^, n (%)**				0.47
0	1 (0.9)	0 (0)	1 (2.2)	
1	46 (41.4)	25 (37.9)	21 (46.7)	
2	41 (36.9)	26 (39.4)	15 (33.3)	
3	23 (20.7)	15 (22.7)	8 (17.8)	
**Interstitial inflammation^†^, n (%)**				0.27
0	2 (1.8)	0 (0)	2 (4.4)	
1	80 (72.1)	49 (74.2)	31 (68.9)	
2	29 (26.1)	17 (25.8)	12 (26.7)	
**Arteriosclerosis^†^, n (%)**				0.44
0	11 (9.9)	5 (7.6)	6 (13.3)	
1	54 (48.6)	35 (53.0)	19 (42.2)	
2	46 (41.4)	26 (39.4)	20 (44.4)	
**Arteriolar hyalinosis^†^, n (%)**				0.02
0	6 (5.4)	1 (1.5)	5 (11.1)	
1	30 (27.0)	15 (22.7)	15 (33.3)	
2	75 (67.6)	50 (75.8)	25 (55.6)	

Data are presented as percent for categorical variables. ^†^ Defined by RPS Diabetic Nephropathy Classification. **Abbreviations:** ESRD, end-stage renal disease; RPS, Renal Pathology Society; IFTA, interstitial fibrosis and tubular atrophy.

**Table 3:** Baseline clinical characteristics of individuals stratified by quartiles of baseline serum transferrin levels

Characteristics	Serum transferrin (g/L)				
	Q1 (n = 26)	Q2 (n = 26)	Q3 (n = 33)	Q4 (n = 26)	*P* value
	≤1.15 g/L	1.15-1.38 g/L	1.38-1.65 g/L	≥1.65 g/L	
Age, mean (SD), y	52 (8)	52 (10)	50 (8)	52 (11)	0.78
Sex, Male, n (%)	9 (34.6)	22 (84.6)	25 (78.1)	16 (59.3)	0.01
Smoking, Never/Ex/Current, (n)	19/3/4	10/5/11	20/5/7	15/7/5	0.14
History of Hypertension, n (%)	26 (100.0)	24 (92.3)	29 (90.6)	26 (96.3)	0.43
BMI, mean (SD), kg/m^2^	25.46 (3.19)	23.21 (2.38)	25.75 (3.74)	25.88 (3.43)	0.06
SBP, mean (SD), mmHg	151 (23)	148 (26)	149 (22)	148 (24)	0.98
DBP, mean (SD), mmHg	90 (13)	86 (12)	87 (12)	85 (10)	0.51
MAP, mean (SD), mmHg	110 (14)	107 (15)	108 (14)	106 (14)	0.75
Duration of diabetes, median (IQR), months	108 (48-132)	114 (24-132)	84 (36-132)	90 (24-180)	0.92
History of DR, n (%)	14 (53.8)	13 (50.0)	22 (68.8)	13 (48.1)	0.51
History of diabetic neuropathy, n (%)	15 (57.7)	17 (65.4)	19 (57.6)	15 (57.7)	0.67
HbA1c, median (IQR), %	6.7 (5.9-8.0)	7.3 (6.7-8.1)	7.2 (6.0-8.4)	7.0 (6.4-7.7)	0.70
FPG, median (IQR), mg/dL	128.45 (83.59-164.11)	133.49 (102.86-210.95)	113.13 (88.45-149.52)	123.85 (99.62-163.03)	0.55
Hemoglobin, mean (SD), g/L	93 (16)	101 (18)	109 (34)	113 (17)	0.01
Serum albumin, mean (SD), g/L	25.2 (5)	27 (5.1)	32.2 (7.1)	39.2 (6.1)	<0.001
CKD stage,1/2/3/4, (n)^ †^	2/3/14/7	3/7/10/6	5/10/10/7	6/3/14/4	0.21
BUN, median (IQR), mg/dL	26.55 (19.27-40.2)	30.69 (21.01-36.69)	21.29 (17.65-36.41)	25.08 (16.53-35.74)	0.75
Serum creatinine, median (IQR), mg/dL	149 (132.8-223)	138 (115-211.8)	123 (102.5-155)	127.5 (76-169)	0.21
eGFR, median (IQR), mL/min/1.73 m^2^	41.9 (29.9-50)	52.3 (36.4-66.5)	58.8 (41-79.8)	53.1 (40.5-77.7)	0.07
24-h proteinuria, median (IQR), g/d	7.14 (4-9.9)	6.4 (3.81-12.56)	4.3 (2.58-7.83)	2.18 (1.07-4.38)	<0.001
Hematuria, n (%)	13 (50.0)	18 (69.2)	19 (59.4)	8 (29.6)	0.04
UA, mean (SD), mg/dL	5.73 (1.37)	6.08 (1.37)	6.5 (1.25)	6.82 (1.49)	0.03
Triglyceride, mean (SD), mg/dL	168.84 (71.98)	156.03 (78.18)	171.03 (137.35)	186.69 (151.71)	0.83
Cholesterol, mean (SD), mg/dL	235.75 (77.93)	202.2 (92.17)	201.46 (44.55)	178.16 (49.86)	0.03
HDL, mean (SD), mg/dL	63.05 (28.54)	56.43 (21.04)	54.11 (19.67)	46.21 (10.52)	0.04
LDL, mean (SD), mg/dL	142.68 (66.27)	124.83 (75.72)	117.53 (37.89)	103.38 (28.75)	0.08
No. of hypertensive drugs, median (IQR)	2 (2-3)	2 (1-2)	2 (1-2)	1 (1-2)	0.12
RAAS inhibitor, n (%)	18 (69.2)	23 (88.5)	24 (75.0)	20 (74.1)	0.39
Statins, n (%)	16 (61.5)	14 (53.8)	19 (59.4)	14 (51.9)	0.93
ESA, n (%)	3 (11.5)	0 (0)	5 (15.6)	0 (0)	0.05
Fe, median (IQR), mmol/L	8.30 (6.10-11.20)	10.14 (9.10-17.30)	12.43 (8.00-17.33)	12.37 (8.90-15.40)	0.01
TIBC, median (IQR), mmol/L	25.14 (22.8-27.4)	31.17 (30.4-32.6)	37.89 (36.4-40.7)	47.4 (45.42-53.07)	<0.001
TSAT, median (IQR), %	33 (24.2-43.1)	32.7 (27.4-54.8)	33.6 (20.1-42.2)	27.3 (18.5-31.7)	0.05
Ferritin, median (IQR), ng/mL	398.25 (197.2-636.5)	394.4 (149.7-607.4)	330 (224.8-581.4)	191.9 (112.5-314.6)	0.03

Data are presented as the mean (standard) for continuous variables with symmetric distribution, median (25^th^-75^th^ percentiles) for continuous variables with asymmetric distribution, or percent for categorical variables. ^†^ CKD stage1: eGFR≥90 mL/min/1.73 m^2^; stage 2: eGFR 60-89 mL/min/1.73 m^2^; stage 3: eGFR 30-59 mL/min/1.73 m^2^; stage 4: eGFR 15-29 mL/min/1.73 m^2^. **Abbreviations:** SD, standard deviation; IQR, interquartile range; BMI, body mass index; SBP, systolic blood pressure; DBP, diastolic blood pressure; MAP, mean blood pressure; DR, diabetic retinopathy; CKD, chronic kidney disease; HbA1c, hemoglobin A1c; FPG, fasting plasma glucose; BUN, blood urea nitrogen; eGFR, estimated glomerular filtration rate; UA, uric acid; HDL, high-density lipoprotein cholesterol; LDL, low-density lipoprotein cholesterol; RAAS, renin-angiotensin-aldosterone system; ESA, erythropoiesis-stimulating agent; TIBC, total iron-binding capacity; TSAT, transferrin saturation; ESRD, end-stage renal disease.

**Table 4:** Pathological characteristics of individuals stratified by quartiles of baseline serum transferrin levels

Characteristics	Serum transferrin (g/L)				
	Q1 (n = 26)	Q2 (n = 26)	Q3 (n = 33)	Q4 (n = 26)	*P* value
	≤1.15 g/L	1.15-1.38 g/L	1.38-1.65 g/L	≥1.65 g/L	
**RPS classification^†^, n (%)**					0.01
I	0 (0)	1 (3.8)	1 (3.1)	2 (7.4)	
IIa	0 (0)	1 (3.8)	5 (15.6)	3 (11.1)	
IIb	0 (0)	2 (7.7)	4 (12.5)	3 (11.1)	
III	20 (76.9)	18 (69.2)	16 (50.0)	11 (40.7)	
IV	6 (23.1)	4 (15.4)	6 (18.8)	8 (29.6)	
**IFTA^†^, n (%)**					0.51
0	0 (0)	2 (7.7)	0 (0)	0 (0)	
1	8 (30.8)	10 (38.5)	18 (56.3)	10 (37.0)	
2	13 (50.0)	10 (38.5)	7 (21.9)	10 (37.0)	
3	5 (19.2)	4 (15.4)	7 (21.9)	7 (25.9)	
**Interstitial inflammation^†^, n (%)**					0.48
0	0 (0)	1 (3.8)	1 (3.1)	0 (0)	
1	18 (69.2)	18 (69.2)	27 (84.4)	17 (63.0)	
2	8 (30.8)	7 (26.9)	4 (12.5)	10 (37.0)	
**Arteriosclerosis^†^, n (%)**					0.17
0	1 (3.8)	3 (11.5)	3 (9.4)	2 (7.4)	
1	10 (38.5)	12 (46.2)	20 (62.5)	12 (44.4)	
2	15 (57.7)	9 (34.6)	9 (28.1)	13 (48.1)	
**Arteriolar hyalinosis^†^, n (%)**					0.50
0	0 (0)	3 (11.5)	1 (3.1)	2 (7.4)	
1	6 (23.1)	10 (38.5)	8 (25.0)	6 (22.2)	
2	20 (76.9)	13 (50.0)	23 (71.9)	19 (70.4)	

Data are presented as percent for categorical variables. ^†^ Defined by RPS Diabetic Nephropathy Classification. **Abbreviations:** ESRD, end-stage renal disease; RPS, Renal Pathology Society; IFTA, interstitial fibrosis and tubular atrophy.

## Abbreviations

T2DM: type 2 diabetes mellitus
DN: diabetic nephropathy
ESRD: end-stage renal disease
HR: hazard ratio
CKD: chronic kidney disease
ESA: erythropoiesis-stimulating agents
RPS: Renal Pathology Society
NDRD: nondiabetic renal diseases
ANCA: anti-neutrophil cytoplasmic antibody
GBM: glomerular basement membrane
BMI: body mass index
RAAS: renin-angiotensin aldosterone system
eGFR: estimated glomerular filtration rate
TIBC: total iron-binding capacity
TSAT: transferrin saturation
SD: standard deviation
IQR: interquartile ranges
LSD: least significant difference
CI: confidence interval
ROC: receiver operating characteristic
AUC: area under the curve
