# Supplementary material for: Serum transferrin predicts end-stage Renal Disease in Type 2 Diabetes Mellitus patients
Source: Int J Med Sci. 2020 Jul 29;17(14):2113–24. doi: 10.7150/ijms.46259 (PMC7484672; doi:10.7150/ijms.46259)
Supplement: Supplementary file 1 — Supplementary figures and tables. [file ijmsv17p2113s1.pdf]

Table S1. Clinical characteristics of individuals for transferrin and iron staining.

Characteristics	Control (n = 10)	DN (n = 20)	*P*-value
Age, mean (SD), y	54 (8)	53 (11)	0.80
Sex, male, n (%)	6 (60.0)	15 (75.0)	0.76
Smoking, Never/Ex/Current, (n)	6/2/2	11/4/5	0.54
BMI, mean (SD), kg/m^2	22.34 (2.2)	26.98 (5.06)	0.05
SBP, mean (SD), mmHg	119 (8)	148 (32)	0.12
DBP, mean (SD), mmHg	84 (6)	83 (17)	0.97
MAP, mean (SD), mmHg	95 (2)	105 (20)	0.23
eGFR, median (IQR), mL/min/1.73 m^2	110 (103.5-116.5)	66.7 (59.9-71.2)	0.04
24-h proteinuria, median (IQR), g/d	0.15 (0.09-0.3)	1.97 (0.82-5.3)	0.01
Fe, median (IQR), μmol/L	16.5 (14.45-17.5)	14.65 (9.39-17.81)	0.01
TIBC, median (IQR), μmol/L	51.9 (49.9-58.5)	38.76 (30.89-40.89)	0.01
TSAT, median (IQR), %	29.7 (25.5-34.1)	36.3 (32.9-40.7)	0.21
Ferritin, median (IQR), ng/mL	191.35 (168.1-210.65)	800.5 (296.85-1182)	0.05
Transferrin, median (IQR), g/L	3.06 (2.96-3.31)	1.52 (1.21-1.6)	0.01

Data are presented as the mean (standard) for continuous variables with symmetric distribution, median (25th-75th percentiles) for continuous variables with asymmetric distribution, or percent for categorical variables.

Abbreviation: SD, standard deviation; IQR, interquartile range; DN, diabetic nephropathy; BMI, body mass index; SBP, systolic blood pressure; DBP, diastolic blood pressure; MAP, mean blood pressure; eGFR, estimated glomerular filtration rate; TIBC, total iron-binding capacity; TSAT, transferrin saturation.

Table S2. Baseline clinical characteristics of the participants for whom iron status information was present or not.

Characteristics	Cohort with complete iron status information (n = 111)	Cohort without iron status information (n = 113)	P value
Age, mean (SD), y	51 (9)	51 (11)	0.92
Sex, male, n (%)	72 (64.9)	68 (60.2)	0.49
Smoking, Never/Ex/Current, (n)	64/20/27	72/17/24	0.64
History of Hypertension, n (%)	105 (94.6)	93 (82.3)	0.01
BMI, mean (SD), kg/m2	25.1 (3.38)	24.8 (4)	0.58
SBP, mean (SD), mmHg	149 (23)	147 (26)	0.50
DBP, mean (SD), mmHg	87 (12)	87 (14)	0.87
MAP, mean (SD), mmHg	108 (14)	107 (16)	0.65
Duration of diabetes, median (IQR), months	96 (36–132)	96 (48–156)	0.35
History of DR, n (%)	62 (55.9)	62 (54.9)	0.34
History of Diabetic neuropathy, n (%)	66 (59.4)	70 (61.9)	0.89
HbA1c, median (IQR), %	6.9 (6.2-8.1)	7.1 (6.2–8.5)	0.69
FPG, median (IQR), mg/dL	126.64 (91.33-169.88)	132.66 (106.2–175.32)	0.73
Hemoglobin, mean (SD), g/L	104 (24)	109 (19)	0.06
Serum albumin, mean (SD), g/L	31 (7.9)	31.8 (7.1)	0.42
CKD stage, 1/2/3/4, (n) †	16/23/48/24	22/24/47/20	0.78
Serum creatinine, median (IQR), µmol/L	136.6 (107–197.6)	125 (87–162)	0.08
eGFR, median (IQR), mL/min/1.73 m^2	51.5 (34.6–66.8)	52.7 (40.3–87)	0.17
24-h proteinuria, median (IQR), g/d	4.64 (2.51-8.37)	5.01 (2.56–9.01)	0.6
Hematuria, n (%)	60 (54.1)	56 (49.6)	0.42
UA, mean (SD), mg/dL	6.3 (1.4)	6.5(1.3)	0.22
Triglyceride, mean (SD), mg/dL	170.67 (115.75)	176.1 (146.5)	0.85
Cholesterol, mean (SD), mg/dL	204.21 (69.66)	209.9 (67.6)	0.42
HDL, mean (SD), mg/dL	54.9 (21.39)	54.5 (18.9)	0.73

LDL, mean (SD), mg/dL	121.82 (55.65)	123.6 (53.2)	0.55
No. of hypertensive drugs, median (IQR)	2 (1–2)	1 (1–2)	0.06
RAAS inhibitor, n (%)	85 (76.6)	84 (74.3)	0.75
Statins, n (%)	63 (56.8)	68 (60.2)	0.58
ESA, n (%)	9 (8.1)	8 (7)	0.74

Data are presented as the mean (standard) for continuous variables with symmetric distribution, median (25th-75th percentiles) for continuous variables with asymmetric distribution, or percent for categorical variables. † CKD stage1: eGFR≥90 mL/min/1.73 m^2; stage 2: eGFR 60-89 mL/min/1.73 m^2; stage 3: eGFR 30-59 mL/min/1.73 m^2; stage 4: eGFR 15-29 mL/min/1.73 m^2.

Abbreviation: SD, standard deviation; IQR, interquartile range; BMI, body mass index; SBP, systolic blood pressure; DBP, diastolic blood pressure; MAP, mean blood pressure; DR, diabetic retinopathy; CKD, chronic kidney disease; HbA1c, hemoglobin A1c; FPG, fasting plasma glucose; BUN, blood urea nitrogen; eGFR, estimated glomerular filtration rate; UA, uric acid; HDL, high-density lipoprotein cholesterol; LDL, low-density lipoprotein cholesterol; RAAS, renin-angiotensin-aldosterone system; ESA, erythropoiesis-stimulating agent.

Table S3. The 5-year renal survival rates according to quartiles of serum iron status parameters.

Iron status parameters	5-year renal survival	*P*- value
Fe		0.09
Q1	7.0%	
Q2	0	
Q3	0	
Q4	14.5%	
Ferritin		0.37
Q1	0	
Q2	20.8%	
Q3	20.8%	
Q4	10.0%	
TIBC		<0.001
Q1	0	
Q2	12.8%	
Q3	0	
Q4	39.8%	
Transferrin		<0.001
Q1	0	
Q2	13.5%	
Q3	7.3%	
Q4	50.9%	
TSAT		0.19
Q1	19.5%	
Q2	0	
Q3	0	
Q4	21.5%	

Abbreviation: TIBC, total iron-binding capacity; TSAT, transferrin saturation.

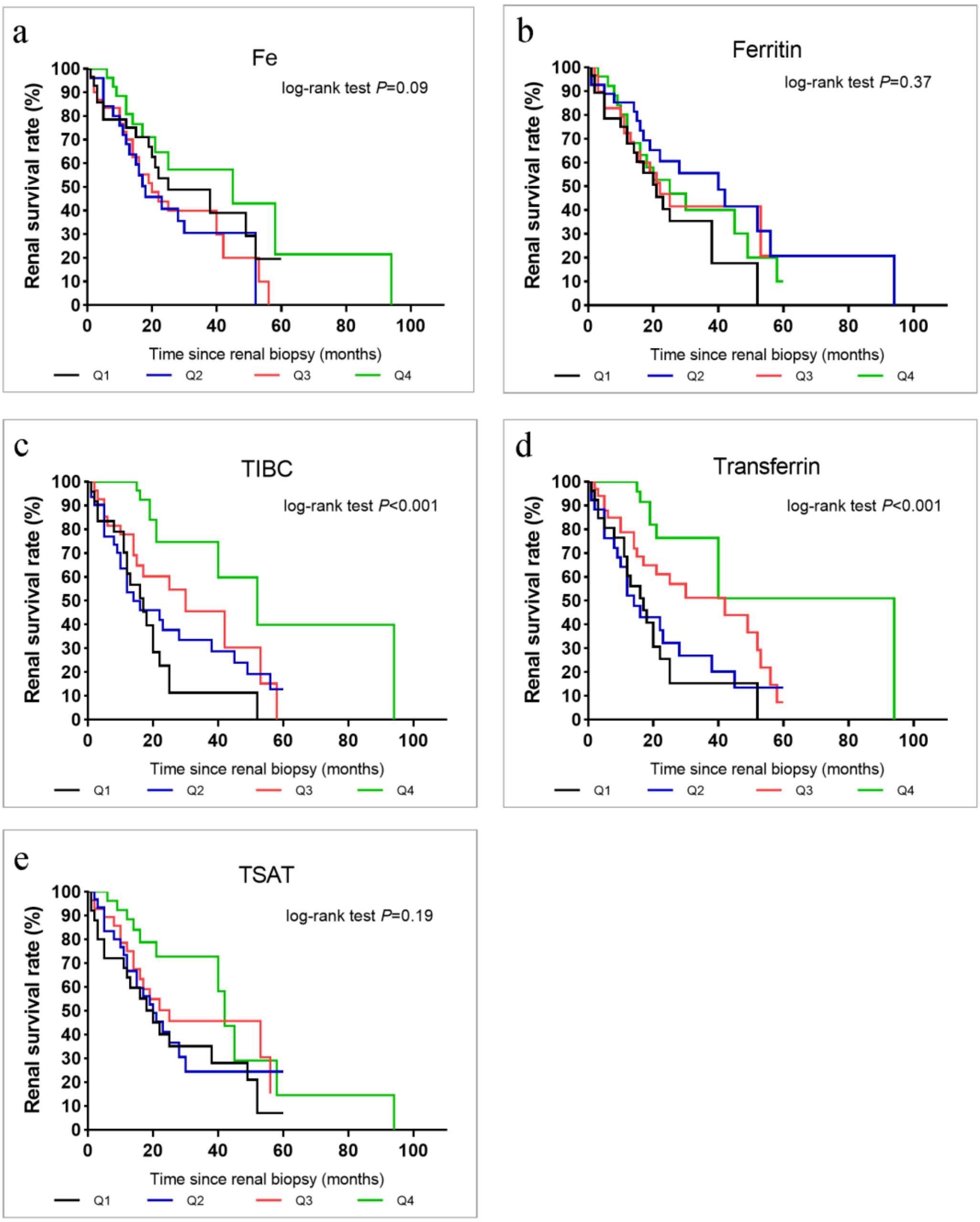

Figure S1. Kaplan-Meier survival curves for renal outcomes according to iron status markers in diabetic patients. (a) Serum iron. (b) Serum ferritin. (c) Total iron-binding capacity. (d) Serum transferrin. (e) Transferrin saturation. TIBC, total iron-binding capacity; TSAT, transferrin saturation.

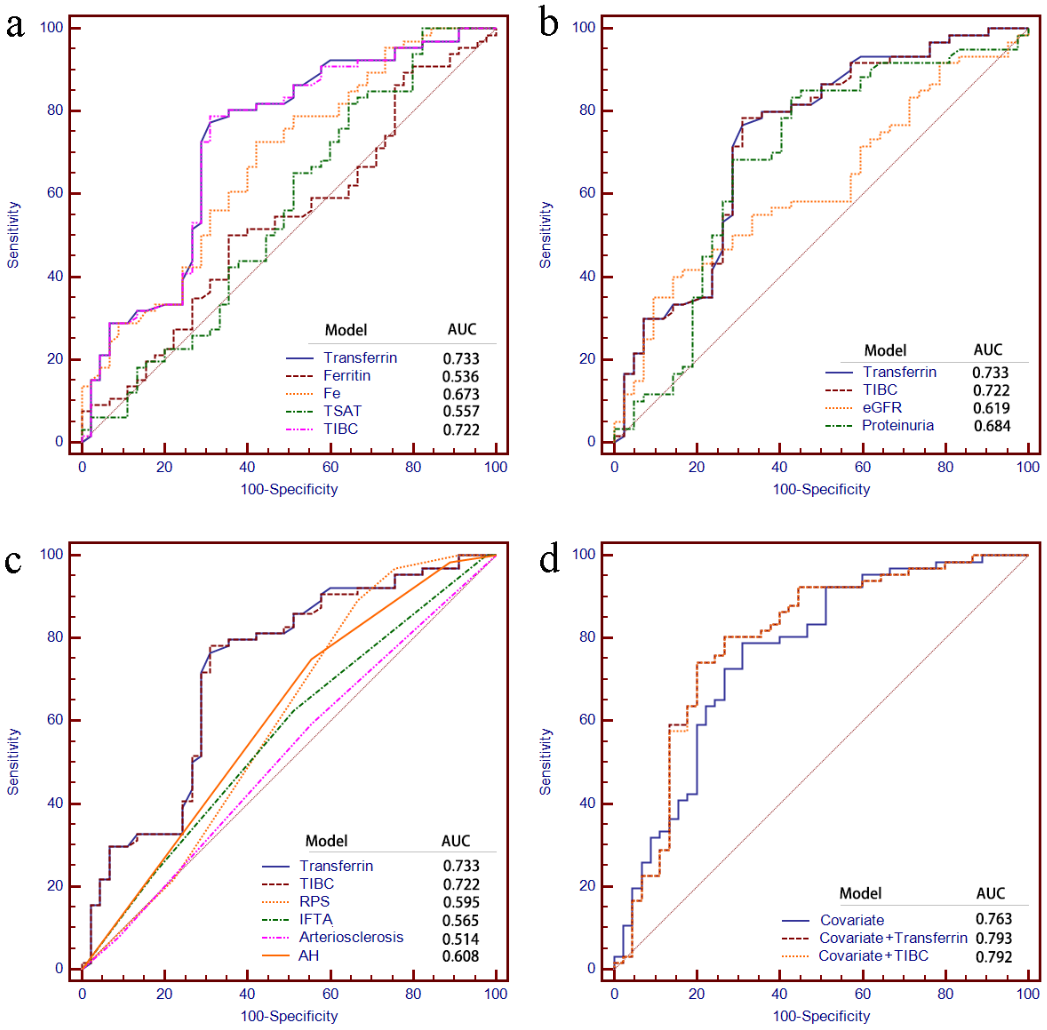

Figure S2. Area under the receiver operating characteristic curve for prediction of ESRD. Serum transferrin had the largest area under the curve (AUC) among the iron status models (a), clinical models (b), pathological models (c) or covariates plus serum transferrin or serum total iron-binding capacity (d). Covariates were eGFR, proteinuria, and hemoglobin. TSAT, transferrin saturation; eGFR, estimated glomerular filtration rate; RPS, Renal Pathology Society; IFTA, interstitial fibrosis and tubular atrophy; AH, artery hyalinosis.

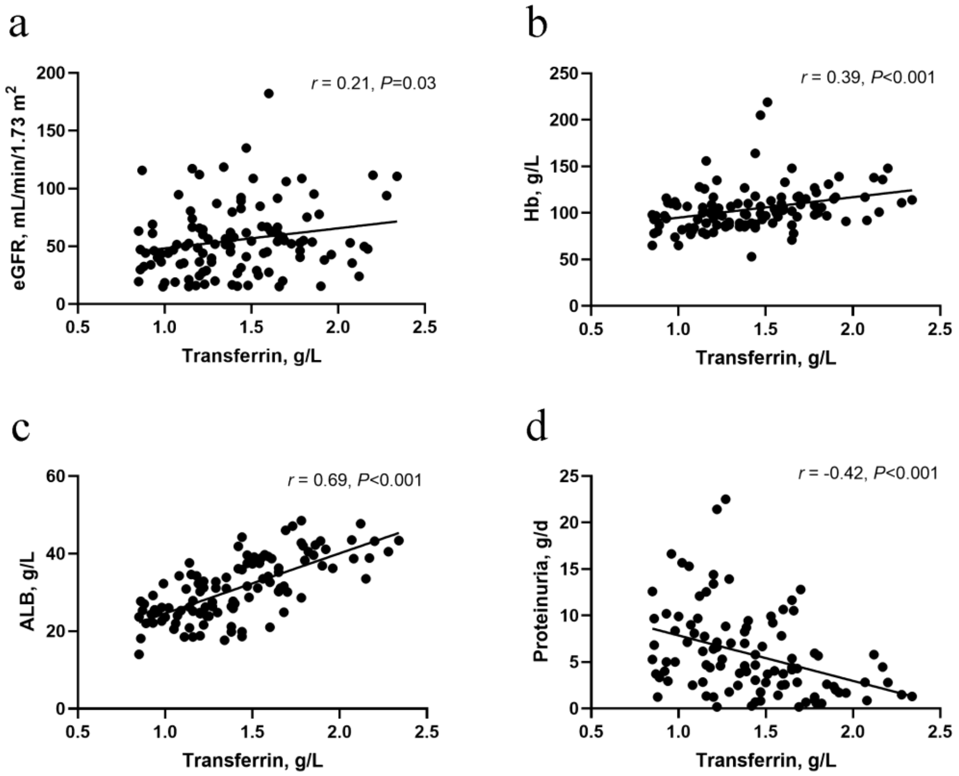

Figure S3. Correlations of baseline serum transferrin levels with (a) estimated glomerular filtration rate (eGFR), (b) serum hemoglobin, (c) serum albumin and (d) proteinuria at the times of renal biopsy among patients with diabetic nephropathy.
